# Preoperative Cannabis Use Associated With an Increased Rate of Reoperation and Postoperative Opioid Use Following Anterior Cervical Decompression and Fusion

**DOI:** 10.7759/cureus.31285

**Published:** 2022-11-09

**Authors:** Jacob Razzouk, Jun H Chung, Wyatt Lindsey, Omar Ramos, Wayne Cheng, Olumide Danisa

**Affiliations:** 1 Medicine, Loma Linda University, Loma Linda, USA; 2 Orthopedic Surgery, Loma Linda University Medical Center, Loma Linda, USA; 3 Biological Sciences, George Washington University, Washington D.C., USA; 4 Orthopedic Surgery, Twin Cities Spine Center, Minneapolis, USA; 5 Orthopedic Surgery, Jerry L Pettis Memorial Veterans Hospital, Loma Linda, USA

**Keywords:** marijuana use, reoperation, anterior cervical decompression and fusion, opioid use, cannabis use

## Abstract

Objectives

The objective of this retrospective cohort study was to evaluate the associations among preoperative cannabis use, postoperative opioid use, and postoperative outcomes following elective anterior cervical decompression and fusion (ACDF).

Methods

Patients who underwent one- or two-level ACDF were characterized preoperatively as active cannabis users, former users, or nonusers. Patients were also classified based on history of preoperative opioid use as chronic users, acute users, or nonusers. Groups were compared based on outcomes including the rate of emergency department visits six months postoperatively, rate of readmissions one year postoperatively, rate of reoperation two years postoperatively, and daily postoperative opioid use measured in milligram morphine equivalents (MMEs) at 0-6 months and 6-12 months postoperatively.

Results

Of the 198 patients included in this study, 13 (6.6%) were active cannabis users, 11 (5.6%) were former users, and 174 (87.8%) were nonusers. The rate of reoperation within two years was 23.1% for active cannabis users, 0% for former users, and 4.0% for nonusers (p*=*0.0075). The average daily opioid use in MMEs 6-12 months postoperatively was 49.4 for active cannabis users, 4.1 for former users, and 13.3 for nonusers (p=0.0014). For chronic opioid users, acute users, and nonusers, the average daily opioid use in MMEs 6-12 months postoperatively was 39.9, 18.4, and 5.7, respectively (p<.0001).

Conclusions

History of cannabis use is associated with increased postoperative opioid use and increased rate of reoperation following elective ACDF.

## Introduction

The use of cannabis is rapidly increasing worldwide [1,2]. It is estimated that 3.9% of the world's population consumes cannabis, which is a population over 10 times the size of consuming cocaine or opioids [1-3]. In the United States and Europe, nationwide estimates of cannabis use are 20% and 26%, respectively [3]. Cannabis is also becoming progressively legalized. As of February 2022, in the United States, 37 states allow cannabis products to be used for medicinal purposes, and 19 states allow recreational cannabis use [4]. In addition to the legalization and use of cannabis becoming more widespread, the potency of cannabis is rising [5]. While therapeutic doses of delta-9-tetrahydrocannabinol (THC) range from 5-20 mg and the average cannabis joint in the 1960s contained 10 mg of THC, the modern joint may contain up to 150 mg of THC [5].

While cannabis has been trialed as a therapeutic agent for several antinociceptive purposes, cannabis use is not without potential risks. Though the extensive effects of cannabis are still not fully understood, cannabis use harbors concerns of harmful addictive, intoxicating, cardiovascular, pulmonary, psychologic, and neurologic effects [6]. Specific to elective spine surgery, Chiu et al. found cannabis abuse was associated with higher rates of perioperative neurologic and respiratory complications, thromboembolism, sepsis, length of hospital stay, hospital charges, and rates of unfavorable discharge disposition [7]. As the prevalence of cannabis use continues to increase globally, understanding cannabis' influences on perioperative and postoperative outcomes following spine surgery is essential for informed patient care [1,2]. Due to its complicated legal status, however, cannabis' influence on spinal surgery outcomes is still not well-understood. The aim of this study was to evaluate the associations among preoperative cannabis use, postoperative opioid use, and postoperative outcomes following elective anterior cervical decompression and fusion (ACDF).

## Materials and methods

Following IRB approval, a retrospective review of the electronic medical record was performed for 198 patients undergoing elective one- or two-level ACDF between 2013 and 2018 at a single tertiary academic center. Exclusion criteria consisted of patients with trauma, neoplasm, three or more levels of fusion, extension of fusion into the occiput or thoracic spine, and spinal fractures. Collected data included indication for surgical intervention, level of the operated cervical spine, American Society of Anesthesiologists (ASA) class, operative time, age and BMI at time of surgery, length of hospital stay, perioperative complications (including myocardial infarction, pulmonary embolism, and deep vein thrombosis), discharge destination (home, skilled nursing facility, rehabilitation), need for readmission or reoperation following ACDF, and medical history including comorbidities and status of opioid and cannabis usage. Patients were classified based on their history of cannabis use at the time of surgery as nonusers, former users, or active users. Patients were also categorized based on the history of preoperative opioid use into chronic opioid users (defined as greater than six months of opioid use preoperatively), acute opioid users (defined as less than six months of opioid use preoperatively), and nonusers. Patients' postoperative opioid use was compared between chronic opioid users, acute users, and nonusers. Collected data used to compare outcomes included the rate of emergency department (ED) visits six months postoperatively, rate of reoperation two years postoperatively, rate of readmissions one year postoperatively, and daily postoperative opioid use measured in milligram morphine equivalents (MMEs) at 0-6 months and 6-12 months postoperatively. 

Statistical analysis

Data collection was performed using Microsoft Excel version 16.58 (Microsoft, Redmond, Washington). SPSS version 28 (IBM Inc., Armonk, New York) was utilized for all subsequent statistical analyses with statistical significance defined as p<0.05. Homoscedasticity was assessed using homogeneity of variance tests and regression residual plots. Q-Q plots and Kolmogorov-Smirnov tests were used to assess for normality of the data. Descriptive statistics utilized means and standard deviations (SD) for demographic and anthropometric data. To assess for correlations between variables, Pearson's correlation tests and enter-method univariate linear regression models were constructed. Correlation coefficients were classified as weak, moderate, and strong, corresponding to value ranges of 0-0.3, 0.3-0.7, and 0.7-1, respectively. Numerical measurement differences among groups were analyzed using one-way analysis of variance (ANOVA) with post hoc Bonferroni and Tukey corrections. Numerical measurement differences between groups were analyzed using independent sample t-tests with Levene's test for equality of variances. Categorical differences between groups were analyzed using Pearson's Chi-square test with Phi and Cramer's V point-biserial correlations. 

## Results

**Figure 1:**
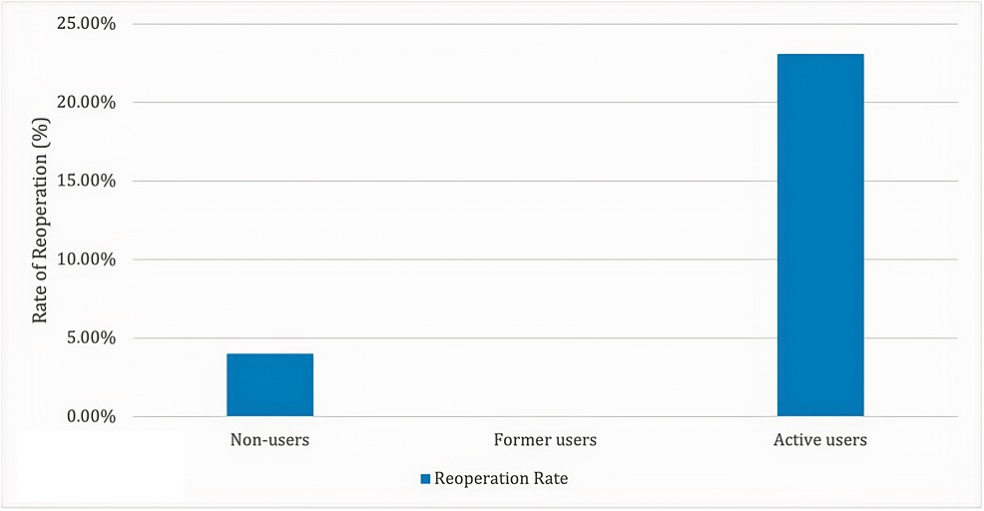
Rate of reoperation two-years postoperatively

**Figure 2:**
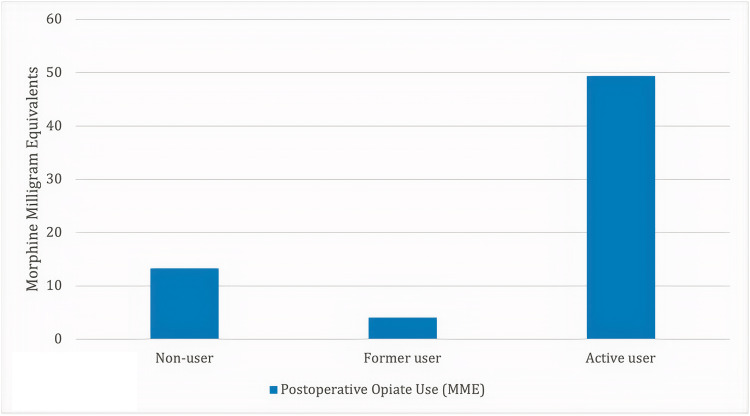
Postoperative opiate use 6-12 months postoperatively

A total of 198 patients were included in this study who received one- or two-level ACDF between 2013 and 2018. Of the 198 patients, 81 patients received one-level ACDF, while 117 received two-level ACDF. The average operative time among all patients was 138 minutes, and the average length of hospital stay was 2.0 days. Myelopathy (n=25), radiculopathy (n=8), spondylosis (n=89), stenosis (n=55), disc herniation (n=13), and degenerative disc disease (n=8) were the listed surgical indications for ACDF. Grouped based on the status of cannabis use, 174 (87.8%) patients did not endorse cannabis use, 11 (5.6%) patients were former cannabis users, and 13 (6.6%) patients were active cannabis users (see Table 1). The rate of reoperation within two years was 4.0% for nonusers, 0% for former cannabis users, and 23.1% for active cannabis users (p=0.0075) (see Figure 1). Rates of ED visits within six months postoperatively were 13.2% for nonusers, 18.2% for former cannabis users, and 15.4% for active cannabis users (p=0.881). The rate of readmissions within one year postoperatively was 12.6% for nonusers, 9.1% for former cannabis users, and 15.4% for active cannabis users (p=0.898). The average daily opioid use in MMEs 0-6 months postoperatively was 94.6 for cannabis nonusers, 58.6 for former cannabis users, and 76 for active cannabis users (p=0.139). The average daily opioid use in MMEs 6-12 months postoperatively was 13.3 for cannabis nonusers, 4.1 for former cannabis users, and 49.4 for active cannabis users (p=0.0014) (see Figure 2). For chronic opioid users, acute users, and nonusers, the average daily opioid use in MMEs 0-6 months postoperatively was 90.9, 89.6, and 92.7, respectively (p=0.957). At 6-12 months postoperatively, these figures decreased to 39.9, 18.4, and 5.7, respectively (p<.0001). No significant correlations were demonstrated with respect to surgical indication, operated cervical level, perioperative complications, discharge destination, readmission or reoperation rates, ethnicity, or ASA class. 

**Table 1:** Patient characteristics stratified by status of cannabis use

	Active user	Former user	Nonuser	p-value
Number of patients	13	11	174	
Age	52.5	49.8	56.3	0.143
BMI	27.7	28.9	30	0.426
Gender (male)	8	6	74	0.324
Gender (female)	5	5	100	

## Discussion

From 2010 to 2020, opioid-related deaths have increased by 225% in the United States alone [8,9]. The severity of the current opioid epidemic has placed an emphasis on decreasing postoperative opioid consumption across all surgical specialties in the United States [10]. However, this effort poses particular difficulty for orthopedic surgeons, among the most frequent opioid prescribers of all surgical and non-surgical specialties [11-13]. Pain management following spinal surgery is especially demanding. With increased postoperative opioid use risking an onset of numerous adverse effects ranging from urinary retention to respiratory depression, there are strenuous demands placed on the spine surgeon to suitably manage postoperative pain while also remaining within acceptable dosing limits [14,15]. Nonetheless, the deliverance of high-quality pain management is a value that should not be compromised. With well-controlled pain management demonstrating strong evidence of improving outcomes, healthcare costs, and patient satisfaction, successful pain management is a vital component of postoperative patient care [16,17]. As opioid prescribers must remain vigilant of the potential addictive and deleterious effects of opioid misuse, a thorough understanding of risk factors for increased postoperative opioid consumption is essential. While the use of cannabis has become common worldwide, the literature has yet to describe the impact of preoperative cannabis usage on postoperative opioid consumption and overall outcomes following ACDF. As the practice of cannabis use continues to grow, knowledge of cannabis' influence on surgical as well as medical decision-making will also need to expand in response to this arising cultural development.

This study is the first to investigate the associations among preoperative cannabis consumption, postoperative opioid use, and overall postoperative outcomes following ACDF. Our findings suggest preoperative cannabis usage is associated with increased postoperative opioid use and increased rate of reoperation following ACDF. These findings are congruent with those of Jamal et al., demonstrating preoperative cannabis use is associated with increased opioid use following inflammatory bowel disease (IBD) surgery [18]. Also, within the context of treating IBD patients, Dalal et al. found preadmission cannabis use was associated with increased inpatient opioid consumption [19]. Outside of the hospital setting, Socías et al. observed a positive relationship between cannabis use and longer retention in opioid agonist treatment (OAT) among those seeking OAT for substance abuse therapy [20]. In a prospective randomized study of patients undergoing elective surgery, Jefferson et al. found those endorsing preoperative cannabis use required significantly higher opioid rescue analgesia in the immediate postoperative period [21]. Assessing patients undergoing various orthopaedic surgeries, Liu et al. found cannabis use is associated with higher postoperative pain scores both at rest and with movement when compared to nonusers, though postoperative opioid use was not assessed [22]. While there remains a dearth of evidence describing the associations between preoperative cannabis use and postoperative factors following spine surgery, in one of few studies comparable to our own, Moon et al. similarly found cannabis use was associated with increased usage of opioids postoperatively following posterior lumbar spinal fusion surgery [23]. However, Moon et al. did not also evaluate the association between cannabis use and postoperative outcomes. While limited research has specifically evaluated the relationship between cannabis and postoperative spinal surgery outcomes, Jennings et al. studied the association between cannabis use and outcomes following total knee arthroplasty [24]. In contrast with our study, Jennings et al. did not find any significant associations [25]. This discrepancy is likely explained by the higher acuity and morbidity associated with patients undergoing spinal surgery, though additional subspecialty-specific investigation of this topic is needed as findings and trends from one subspecialty may not necessarily generalize across other subspecialties.

An exact mechanism explaining why cannabis may be associated with increased opioid needs postoperatively remains undetermined. However, it is known that the mechanism of action by which cannabis exerts its effects is via G protein-coupled (GPC) binding of THC and cannabidiol (CBD) to cannabinoid receptors 1 and 2 (CB1 and CB2 ) [25,26]. THC is a partial agonist of CB1, and CBD is a CB1 receptor-negative allosteric modulator [27]. CB1 receptors modulate nociceptive processing in the hippocampus, cerebellum, and basal ganglia and are similar in neurochemical structure to opioid receptors [25,26,28]. Sensory neurons in the dorsal root ganglion contain CB2 receptors that also serve as central sites of nociceptive integration. Activation of the cannabis GPC pathway stimulates the endogenous cannabinoid system (ECS), which results in various neuromodulatory effects such as the inhibited release of gamma-aminobutyric acid (GABA), glutamate, norepinephrine, and acetylcholine [28-30]. ECS also results in increased release of dopamine and endocannabinoids, endogenous neuroactive messengers that influence reward and pain pathways [25,26,28]. One hypothesis described by Moon et al. proposes that increased postoperative opioid use is associated with preoperative cannabis use due to cannabis users self-medicating for increased preoperative pain compared to nonusers [23].

Regardless of the exact theory or root cause that may potentially explain an association between preoperative cannabis use and postoperative opioid use, our results suggest the utility of completing a thorough preoperative history on elective ACDF patients that includes cannabis use. As preoperative cannabis status may be a risk factor for an increase in rate of reoperation and postoperative opioid needs, spine surgeons should be well-informed of their patient's history of cannabis use as well as cannabis' general influences on perioperative and postoperative outcomes.

Limitations

This study is not without several limitations. Its retrospective, single-institution-based nature is a potential limitation of its generalizability. Furthermore, this study may be limited by inter-surgeon differences in ACDF technique that may cause subsequent variations in ACDF outcomes. An added limitation was our inability to assess the recency and quantity of cannabis consumed by patients either preoperatively or postoperatively and whether or not all opioids prescribed to the patients in this study were actually consumed. Moreover, patient history of cannabis use may be potentially underinflated due to patients feeling uncomfortable in disclosing their use of cannabis due to stigma-related or legal concerns. Despite these limitations, we hope this study motivates future research to further inform the literature in light of growing opioid and cannabis consumption worldwide.

## Conclusions

This study suggests preoperative opioid and cannabis use are associated with increased opioid use 6-12 months following ACDF. Active, preoperative cannabis may be associated with an increased rate of reoperation within two years following ACDF. As an endorsement of preoperative cannabis use may be a risk factor for an increase in the rate of reoperation and postoperative opioid needs, spine surgeons should be well-informed of their patient's history of cannabis use as well as cannabis' general influences on perioperative and postoperative outcomes. As such, our study suggests the utility of completing a thorough preoperative history on elective ACDF patients that include inquiries regarding the status of cannabis use.
